# Chronic morphine regulates TRPM8 channels via MOR-PKCβ signaling

**DOI:** 10.1186/s13041-020-00599-0

**Published:** 2020-04-14

**Authors:** Mircea Iftinca, Lilian Basso, Robyn Flynn, Charlie Kwok, Corinne Roland, Ahmed Hassan, Manon Defaye, Rithwik Ramachandran, Tuan Trang, Christophe Altier

**Affiliations:** 1 Department of Physiology and Pharmacology, Inflammation Research Network-Snyder Institute for Chronic Diseases and Alberta Children’s Hospital Research Institute, University of Calgary, 3330 Hospital Dr NW, Calgary, Alberta T2N 4N1 Canada; 2 Hotchkiss Brain Institute. Cumming School of Medicine. University of Calgary, Calgary, Alberta T2N 4N1 Canada; 3 Department of Physiology and Pharmacology. Schulich School of Medicine & Dentistry, University of Western Ontario, London, ON N6A 5C1 Canada

**Keywords:** Transient receptor potential channel subfamily M (melastatin) member 8 (TRPM8), Mu opioid receptor (MOR), Morphine, Protein kinase C beta (PKCβ), Dorsal root ganglia (DRG) neurons, Cold hyperalgesia

## Abstract

Postoperative shivering and cold hypersensitivity are major side effects of acute and chronic opioid treatments respectively. TRPM8 is a cold and menthol-sensitive channel found in a subset of dorsal root ganglion (DRG) nociceptors. Deletion or inhibition of the TRPM8 channel was found to prevent the cold hyperalgesia induced by chronic administration of morphine. Here, we examined the mechanisms by which morphine was able to promote cold hypersensitivity in DRG neurons and transfected HEK cells. Mice daily injected with morphine for 5 days developed cold hyperalgesia. Treatment with morphine did not alter the expressions of cold sensitive TREK-1, TRAAK and TRPM8 in DRGs. However, TRPM8-expressing DRG neurons isolated from morphine-treated mice exhibited hyperexcitability. Sustained morphine treatment in vitro sensitized TRPM8 responsiveness to cold or menthol and reduced activation-evoked desensitization of the channel. Blocking phospholipase C (PLC) as well as protein kinase C beta (PKCβ), but not protein kinase A (PKA) or Rho-associated protein kinase (ROCK), restored channel desensitization. Identification of two PKC phosphorylation consensus sites, S1040 and S1041, in the TRPM8 and their site-directed mutation were able to prevent the MOR-induced reduction in TRPM8 desensitization. Our results show that activation of MOR by morphine 1) promotes hyperexcitability of TRPM8-expressing neurons and 2) induces a PKCβ-mediated reduction of TRPM8 desensitization. This MOR-PKCβ dependent modulation of TRPM8 may underlie the onset of cold hyperalgesia caused by repeated administration of morphine. Our findings point to TRPM8 channel and PKCβ as important targets for opioid-induced cold hypersensitivity.

## Introduction

Opioids are widely used analgesics for the treatment of moderate to severe pain but their use often leads to the development of severe side effects, including opioid induced cold hyperalgesia [1–3]. The clinically used analgesic morphine acts on MOR expressed on the primary afferent pathway. At the cellular level, MOR activates Gα_i/o_ proteins leading to inhibition of cyclic adenosine monophosphate (cAMP) generation, increased extracellular signal–regulated kinase (ERK) phosphorylation, inhibition of presynaptic voltage-gated calcium channels at primary afferent central terminals and activation of postsynaptic G protein–regulated inwardly rectifying potassium channels (GIRKs) in projecting neurons of the spinal dorsal horn [4, 5]. Although there is still controversy as to whether morphine promotes analgesia by acting on peripheral rather than central loci of MOR [6, 7], there is evidence that peripheral MOR in DRG contributes to opioid-induced hyperalgesia.

Cold hyperalgesia can appear in diseased states such as complex regional pain syndrome, diabetic neuropathy or chronic nerve injury [8]. Change in cold perception is also a common manifestation of chronic morphine administration and associated withdrawal syndromes [1, 9]. One major ion channel that transduces cold in sensory DRG neurons is the TRPM8, which is present in peptidergic neurons and can sense temperature changes in the range of innocuous cold (15–28 °C) [10–12]. TRPM8 is activated by chemical cooling agents such as menthol, but inhibited by inflammatory agents [13, 14]. TRPM8 knockout mice show impaired cold sensation, cold allodynia, and analgesia, demonstrating the role of TRPM8 in cold sensation [15–17].

A functional link between MOR and TRPM8 has been suggested to support the well-recognized opioid-induced modulation of thermosensation, but results are conflicting [18]. Whereas some authors have shown that prolonged MOR stimulation leads to the internalization of TRPM8 thereby causing morphine-induced cold analgesia [18], others have observed that chronic administration of morphine could lead to an upregulation of TRPM8 expression in sensory neurons [19]. Here, we examined the signaling pathway(s) linking MOR to TRPM8, using behavioral, electrophysiological and biochemical assessment of TRPM8 function and expression after chronic morphine treatment. Our results provide insight into the molecular mechanisms of opioid-induced cold hypersensitivity.

## Methods

### Expression vectors

Human TRPM8 cDNA was a gift from Dr. Ramachandran. TRPM8-YFP was cloned into pEYFP-N1 using AfeI/XhoI restriction sites. Phosphorylation sites were identified using PhosphoSitePlus (https://www.phosphosite.org/homeAction). Mutations of the two TRPM8 serine residues 1040 and 1041 were done using site-directed mutagenesis; the mutations were verified by sequencing and subcloned into pcDNA3 vector to eliminate undesirable mutations elsewhere in the vector. Rat MOR was previously described [20]. The PKC-βII-GFP was a gift from Dr. S. Ferguson. The PAR2 was a gift from Dr. M. Hollenberg. CB1, α1AR and α2AR plasmids were obtained from the cDNA resource center (https://www.cdna.org/faq.html). Constructs were confirmed with DNA sequencing (U of Calgary core DNA service).

### Cell culture and transient transfection

Human embryonic kidney (HEK) 293 tsA-201 cells were grown to 80% confluence at 37 °C (5% CO_2_) in Dulbecco’s modified Eagle’s medium (+ 10% fetal bovine serum, 200 units/ml penicillin and 0.2 mg/ml streptomycin (Invitrogen, Carlsbad, CA, USA)). Cells were dissociated with trypsin (0.25%)-EDTA before plating on glass coverslips (for electrophysiology and calcium imaging) or plastic tissue culture plates (for biochemistry). Cells were transfected with 1.5 μg of TRPM8 and 1 μg of MOR using the calcium phosphate method. For electrophysiology, 0.3 μg of green fluorescent protein (GFP) was added as a transfection marker. Cells were washed 8 h after transfection; electrophysiological recordings or calcium imaging were conducted 24 h later while biochemistry was performed 48 h after transfection.

### Isolation of DRG neurons

DRG neurons were excised from 6 week old mice and enzymatically dissociated in Hank’s balanced salt solution (HBSS) containing 2 mg/ml collagenase type I and 4 mg/ml dispase (Invitrogen) for 45 min at 37 °C. DRGs were rinsed twice in HBSS and once in Neurobasal A culture medium (Thermo Fisher Scientific) supplemented with 2% B-27, 10% heat-inactivated fetal bovine serum (HI-FBS), 100 μg/ml streptomycin, 100 U/ml penicillin, 100 ng/ml of Nerve Growth Factor (NGF) and 100 ng/ml glial cell-derived neurotrophic factor (GDNF) (all from Invitrogen). Individual neurons were dispersed by trituration through a fire-polished glass Pasteur pipette in 4 ml media and cultured overnight at 37 °C with 5% CO2 in 96% humidity on glass coverslips previously treated 25% Poly-Ornithine and Laminin (both from Sigma).

### Biotinylation assay

Cells were passaged onto 60 mm plate and transfected with TRPM8-YFP. Forty eight hours after transfection, surface proteins were biotinylated using 0.75 mg/ml EZ Link Sulfo-NHS-SS biotin (Invitrogen) in HBSS, then quenched with 100 mM glycine. Cells were then harvested and lysed in RIPA (0.1% SDS, 1% Triton X-100 and 0.5% Na deoxycholate in PBS) with HALT protease inhibitor (Invitrogen) for 45 min. Protein concentration of lysates was determined using the Bradford assay and 1 mg of lysate was precipitated on Neutravidin beads (Thermo Fisher) for 2 h. Beads were washed in HBSS and bound proteins eluted in 4X Laemmli buffer (200 mM Tris, 8% SDS, 40% glycerol, 400 mM beta-mercaptoethanol, and 0.04% Coomassie blue). Biotinylated proteins were then separated by SDS-PAGE, transferred to nitrocellulose membranes and probed for GFP using rabbit GFP antibody (Chromotek; 1:5000). The membrane protein Na+/K+ ATPase (Cell Signaling antibody) was used for normalization of TRPM8 signal.

### Co-immunoprecipitation and western blotting

Cells were harvested 48 h after transfection with 7.5 μg of TRPM8-HA, PKCβII-GFP and MOR cDNA and lysed in RIPA (0.1% SDS, 1% Triton X-100 and 0.5% Na deoxycholate in PBS) with Halt protease inhibitor (Thermo Scientific) and phosphatase inhibitor (Thermo Scientific) for 20 min. Cell lysates were precipitated with the ChromoTek GFP-Trap® coupled to agarose beads and incubated with overnight rotation at 4 °C. Beads and precipitates were washed 3x in RIPA then eluted from beads in 1x Laemmli buffer. Samples were run on 8% tris-glycine gels and transferred to nitrocellulose membranes, then probed with rabbit anti-GFP (Torrey Pines) 1:5000 or the monoclonal anti HA (Covance) followed by ECL-optimized anti-rabbit (GE Healthcare).

### RNA extraction and RT-qPCR

DRGs were harvested after control or prolonged morphine treatment, dissociated using a bullet blender (Next Advance) with SSB02 beads (Next Advance) in RLT buffer (Qiagen, Toronto, Ontario, Canada). Total RNA was extracted using an RNeasy Mini kit (Qiagen), according to the manufacturer’s instructions. The quality and quantity of RNA were determined using a Nanodrop 2000c spectrophotometer (Thermo-Fisher Scientific, Montréal, Quebec, Canada). Relative MOR, TRPM8, Potassium channel subfamily K member 4 (TRAAK) and Potassium channel subfamily K member 2 (TREK-1) gene expresion (normalized to Ribosomal Protein Lateral Stalk Subunit P1 (RPLP)) was determined by qPCR using BrightGreen PCR Master Mix (ABMgood) and a StepOnePlus real-time PCR detection system (Applied Biosystems, Burlington, Ontario, Canada). The primers used are included in the Supplementary Table 1 and were designed to amplification of DNA.

### Calcium imaging

Transfected HEK cells or isolated DRGs were loaded with Fura-2-AM (0.5 μM for 30 min, Invitrogen) before imaging. The recordings were performed at 37 °C. We perfused either menthol (100 μM, Sigma) or extracellular solution at 25 °C, at a rate of ~ 1 ml/min, to examine TRPM8 activity in control conditions or in cells treated with morphine (500 nM) for 16 h. Images of cell-field were continuously recorded every 100 ms using 340 and 380 nm excitation wavelengths with emission measured at 520 nm with a microscope based imaging system (Olympus IX73) on CellSense software. Images were processed using ImageJ by drawing discrete regions of interest around cells that responded to menthol or cold. We expressed a change in fluorescence as a percentage change from the amplitude of the first response to menthol or cold.

### Behavior analysis

6-week old C57BL/6 male mice were obtained from Jackson Laboratory (USA) and were acclimated for 2 days prior to behavioral experiments. Mice were housed with free access to food and water, with a 12/12 light dark cycle. All experiments were conducted on age-matched animals, under protocols approved by the University of Calgary Animal Care Committee and in accordance with the international guidelines for the ethical use of animals in research and guidelines of the Canadian Council on Animal Care.

Behavioral assessment of cold sensitivity in mice was done using the cold plate test. Mice received intraperitoneal injection of escalating doses of morphine (from 10 mg/kg to 50 mg/kg) or saline, twice daily for 5 days. Cold sensitivity was measured using the cold plate test (Bioseb). Briefly, mice were placed on the surface of a metal plate cooled at 0 °C (with an ambient temperature of 21 °C). The time taken for each mouse to show the first nociceptive response (paw withdrawal, shaking, liking of the rear paw, or jumping to try to escape) was monitored on the first and last day of the morphine injection. An experimenter blinded to the treatments performed the behavioral assessment. Data points represent each individual mouse.

### Electrophysiological measurements

Electrophysiological recordings were conducted using an external solution containing (in mM): 140.0 NaCl, 1.5 CaCl_2_, 2.0 MgCl_2_, 5.0 KCl, 10.0 HEPES, 10.0 D-glucose, pH 7.4 adjusted with NaOH. HEK cells expressing the transfected TRPM8 channel and MOR were identified via GFP fluorescence using an inverted epi-fluorescence microscope (Olympus IX51, Olympus America Inc., USA). DRG neurons were recorded based on size, knowing that TRPM8 is expressed in a subpopulation of small neurons [21]. Membrane currents were measured using conventional whole-cell patch clamp and action potentials were recorded using current clamp. Borosilicate glass (Harvard Apparatus Ltd., UK) pipettes were pulled and polished to 2–5 MΩ resistance with a DMZ-Universal Puller (Zeitz-Instruments GmbH., Germany). For the voltage clamp experiments pipettes were filled with an internal solution containing (in mM): 120.0 CsCl, 10.0 EGTA, 10.0 HEPES, 3.0 MgCl_2_, 2.0 ATP Na2, 0.5 GTP, pH 7.2 adjusted with CsOH whereas for the current clamp experiments the internal solution contained (in mM): 140.0 KCl, 5.00 NaCl, 1 CaCl_2_, 1.0 EGTA, 10.0 HEPES, 1.0 MgCl_2_, 3.0 ATP Na2, pH 7.3 adjusted with KOH. All solutions were prepared and used at room temperature (22 ± 2 °C) and their osmolarity adjusted to 310 mOsm. Data obtained with different types of internal solutions were not pooled. For the current clamp experiments the spontaneous activity of the DRG neurons was recorded at room temperature (~ 22 °C) for 3 min before application of the first cold (10 °C) or menthol challenge (applied to the bath at ~ 1000 μm from the cell at a rate of 500 μl/min). Only the neurons in which the resting membrane potential was more negative than − 40 mV were used. Recordings were performed using an Axopatch 200B amplifier (Axon Instruments, Foster City, CA, USA). Voltage- and current-clamp protocols were applied using pClamp 10.4 software (Axon Instruments). Data were filtered at 1 kHz (8-pole Bessel) (whole cell voltage clamp) and 5 kHz (current clamp) and digitized at 10 kHz with a Digidata 1550 A converter (Axon Instruments). Average DRG neurons capacitance was 11.26 ± 0.69 pF for naive and 10.59 ± 0.98 pF for morphine-treated animal. HEK cells had an average capacitance of 26.78 ± 2.15 pF. Only the cells that exhibited a stable voltage control throughout the recording were used for analysis.

For prolonged morphine treatment, neurons were incubated with a low concentration of morphine (500 nM) for 16 h.

### Chemicals and drugs

Menthol, DAMGO, morphine sulfate, phorbol 12-myristate 13-acetate (PMA) and Y27632 were obtained from Sigma-Aldrich. The PKC inhibitor GF109203X, and TRPM8 antagonist AMTB (N-(3-Aminopropyl)-2-[(3-methylphenyl) methoxy]-N-(2-thienylmethyl) benzamide hydrochloride) and H89 were purchased from Tocris Bioscience. The specific PKCβ blocker Enzastaurin (LY317615) was purchased from Selleckchem and Naloxone from Santa Cruz Biotechnology.

### Statistics

Data analysis and offline leak subtraction were completed in Clampfit 10.4 (Axon Instruments), and all electrophysiology curves were fitted using Origin 7.0 analysis software (OriginLab, Northampton, MA, USA). The menthol and cold dose-response relationships were fitted using the Hill equation. All averaged data are plotted as mean ± SEM and numbers in parentheses reflect the number of cells (n). The Shapiro-Wilk normality test was used for our two or multiple group comparison. Statistical analyses were completed with Origin 7.0 software using unpaired t-tests, or the nonparametric Mann-Whitney U test, when data were compared from two independent groups of cells. One-way analysis of variance (ANOVA) followed by the Bonferroni or Sidak’s post hoc test, or the nonparametric Kruskal-Wallis ANOVA test, was used for multiple comparisons, with the criterion for statistical significance set at *p* < 0.01.

## Results

### Chronic morphine treatment induces cold hypersensitivity and enhances the excitability of TRPM8-expressing neurons

**Fig. 1:**
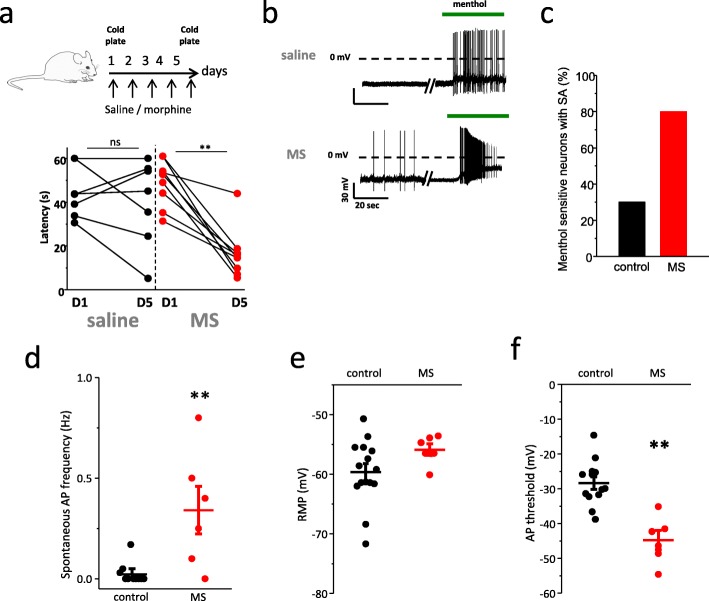
Chronic morphine treatment promotes cold hypersensitivity by enhancing the excitability of menthol-sensitive neurons. **a** Top, illustration representing the experimental paradigm of morphine treatment in mice. Saline or morphine injections were performed twice a day for 5 days. Cold sensitivity was measured using the cold plate test. Bottom graph shows the latency of the first nociceptive response (paw withdrawal, licking, shaking, or jumping to try to escape) to a cold stimulus. Each circle represents the latency of an individual mouse treated with saline (black) or morphine (red) and recorded on the first and last day of injection. Statistical analysis was performed using Two-Way ANOVA followed by Sidak’s post hoc test (**p < 0.001; D1 compared to D5, *n* = 7 mice per group). **b** Representative current-clamp recordings showing the menthol-induced depolarization (menthol: 100 μM) and AP firing of DRG neurons from saline or morphine-treated mice (MS). **c** The percentage of menthol-responsive small diameter neurons exhibiting spontaneous activity was significantly increased between saline (black, 30%; *n* = 14) and morphine (red, 80%; *n* = 16) groups (from 5 and 6 mice respectively). **d** Spontaneous AP frequency in menthol-responsive neurons from saline (black: 0.03 ± 0.02 Hz, *n* = 10) versus morphine-treated mice (red: 0.34 ± 0.12 Hz, *n* = 6). **e** Resting membrane potential of menthol-responsive neurons from vehicle and morphine-treated mice (59.6 ± 1.4 mV vs. 55.9 ± 0.98 mV, *n* = 15 and 17, respectively). **f** Action potential threshold of menthol-responsive neurons from vehicle and morphine-treated mice (28.4 ± 1.77 mV vs. 44.75 ± 2.73 mV, *n* = 13 and 6, respectively). Error bars indicate ±SEM. Statistical analysis was performed using unpaired t-test and the Mann-Whitney test in (**d**) (**p < 0.05)

To determine whether morphine treatment could modulate cold sensitivity, we used a behavioral paradigm in which cold response latencies were assessed on the first and fifth day of morphine injection [22] (see Methods). Mice treated daily with morphine exhibited cold hyperalgesia relative to saline control groups (Fig. 1a). Following behavior testing, DRGs were harvested and the neurons isolated for electrophysiological and calcium imaging recordings. In current clamp configuration, we found that menthol sensitive neurons exhibited both increased spontaneous activity and menthol-evoked action potential discharge after morphine treatment (Fig. 1b). The percentage of menthol-sensitive small diameter neurons exhibiting spontaneous activity was enhanced (Fig. 1c) as was the frequency of spontaneous activity (Fig. 1d). Chronic morphine did not change significantly the neuronal resting membrane potential but lowered the action potential threshold (Fig. 1e and f). To assess whether morphine signaling was affecting voltage-gated ionic conductances, we measured the neuronal activity evoked by ascending ramps of injected current. Neurons from morphine treated mice exhibited an increase in the frequency of AP compared to control (Fig. S1a and b). Overall, these data indicate that chronic exposure to morphine enhances the excitability of TRPM8-expressing neurons and leads to a modulation of voltage-gated ion channels.

### Chronic morphine reduces both cold and menthol-evoked desensitization of TRPM8

**Fig. 2:**
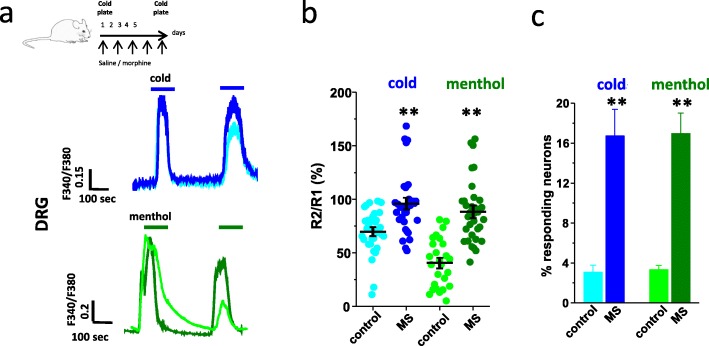
Chronic treatment with morphine prevents activation-induced desensitization of TRPM8. **a** Top, illustration representing the experimental paradigm of morphine treatment in mice as in Fig. 1. Bottom, representative calcium imaging traces to two repeated applications of cold (20 °C, blue) or menthol (100 μM, green) in DRG neurons isolated from saline- (light color) and morphine- (MS; dark color) treated mice. **b** Whisker plots showing the mean ratio (R2/R1) of the amplitude response to cold stimulation in control (69.65 ± 3.2%; light blue) vs morphine (95.85 ± 4.96%; dark blue) treated mice (*n* = 33 and 33, respectively) or menthol stimulation in control (40.57 ± 4.44%; light green) vs morphine (88.25 ± 5.09%, dark green) treated mice (*n* = 30 and 33, respectively). **c** Percentage of responding neurons, out of all recorded neurons, to the first application of cold (3.06 ± 0.71% [36/1200] in control (light blue) vs 16.71 ± 2.68% [326/1916] after morphine (dark blue); *n* = 7 and 5 independent experiments, respectively) or menthol (3.31 ± 0.44% [37/1138] in control (light green) vs 16.95 ± 2.05% [32/1916] after morphine (dark green), *n* = 4 and 8 independent experiments, respectively). Error bars indicate ±SEM. Statistical analysis was performed using the Kruskal-Wallis ANOVA (**b**) or One-Way ANOVA followed by Bonferroni post hoc test (**p < 0.05)

TRPM8 is the primary cold receptor of the somatosensory system and its desensitization has been reported to account for the adaptation to environmental cold [23]. We thus assessed TRPM8 desensitization after morphine treatment by measuring TRPM8 calcium responses to two repeated stimulations with either cold or menthol. In DRG neurons from saline injected animals, TRPM8 calcium signals were markedly desensitized in response to a second stimulation by cold or menthol [23, 24]. In contrast, DRG neurons from morphine treated mice showed a loss of TRPM8 desensitization to both stimuli (Fig. 2a and b). As the desensitization of TRPM8 in response to the second stimulation is a calcium sensitive process that is directly contingent on the intracellular calcium load evoked by the first stimulation, we asked whether morphine was able to affect the first response to cold or menthol. As shown in Fig. S1d, the response to cold or menthol did not differ in cultured sensory neurons exposed to morphine or in neurons isolated from morphine- treated mice, relative to control. In accordance with previous published results [12, 21, 25, 26], we found that cold sensitive DRG neurons were a rare subset of small diameter neurons (< 20 μm in diameter). In control mice, 3.06% of total neurons (37/1200) responded to cold and 3.31% (37/1138) to menthol (100 μM). Interestingly, this number increased in morphine-treated groups, with 16.71% of total neurons (304/1822) responding to cold and 16.95% (325/1916) to menthol (Fig. 2c**)**. These results indicate that chronic morphine caused a subset of menthol-insensitive neurons to acquire de novo responsiveness to menthol and cooling. Nonetheless, no significant change in mRNA expression was found for either TRPM8 or other cold sensitive TREK-1 and TRAAK channels (Fig. S1c), implying a functional rather than transcriptional regulation of these neurons [11, 26–28].

### Sustained morphine treatment enhances TRPM8 responsiveness to cold and menthol in cultured DRG neurons

**Fig. 3:**
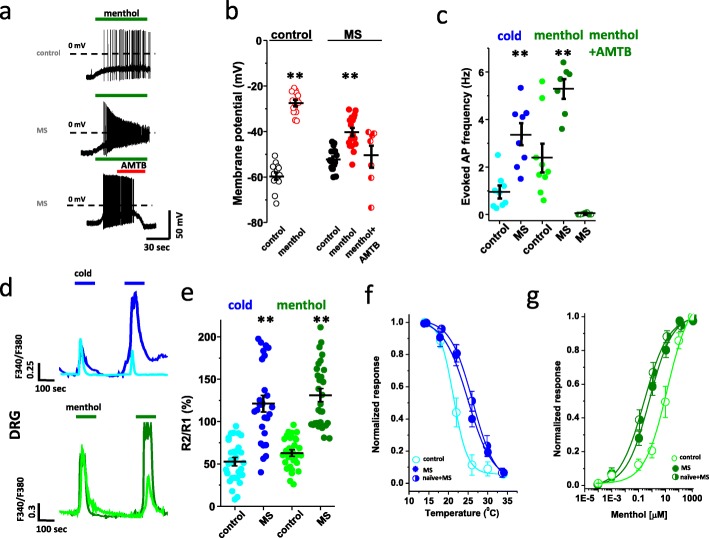
Morphine enhances TRPM8 sensitivity to cold and menthol. **a** Representative whole cell current**-**clamp recording of menthol-evoked depolarization and AP firing (menthol: 100 μM) of DRG neurons collected from naïve mice and exposed to morphine (MS) overnight versus control. **b** Whisker plots of the membrane potential of menthol sensitive neurons exposed to vehicle or morphine, before (dark) or after (red) menthol stimulation (− 59.6 ± 1.4 mV vs. -27.6 ± 1.2 mV in control, *n* = 15; − 52.3 ± 1.2 mV vs. -40.2 ± 1.2 mV with morphine, *n* = 17), or menthol+AMTB (− 51.9 ± 1.9 mV; *n* = 7). **c** Whisker plot of the AP discharge, measured over a 1 min. Application of cold (0.95 ± 0.24 Hz in control (light blue) vs 3.6 ± 0.65 Hz with morphine (dark blue), *n* = 9 and 8, respectively) or menthol (2.4 ± 0.57 Hz in control (light green) vs 5.29 ± 0.39 Hz with morphine (dark green), *n* = 9 and 7, respectively), or menthol + AMTB (0.026 ± 0.01 Hz; *n* = 7). **d** Representative calcium imaging traces to two repeated applications of cold (20 °C, blue) or menthol (100 μM, green) in cultured DRG neurons exposed to morphine (500 nM) overnight. **e** Whisker plots showing the mean ratio (R2/R1) of the amplitude response to cold (52.85 ± 23.72% in control, light blue, vs 121.07 ± 45.89% with MS, dark blue; *n* = 60 and 54, respectively) or menthol (62.84 ± 17.87% in control, light green, vs 130.81 ± 37.48% with MS, dark green; *n* = 64 and 58, respectively) responses illustrated in (**d**). **f** Temperature-response curve measured by calcium imaging during cooling ramp on DRG neurons from naive (median temperature value 21.29 ± 0.53 °C, control, *n* = 36) versus morphine-treated mice (median temperature value, 26.23 ± 0.78 °C, in vivo MS, *n* = 41) or naïve cultured DRG neurons exposed to morphine overnight (median temperature value, 24.98 ± 0.57 °C, in vitro MS, *n* = 28). Sensitization of the temperature-response relationship is indicated by the shift toward warmer temperature. **g** Dose-response curve evoked by menthol, measured by calcium imaging on DRG neurons from naive (EC50 = 13.86 ± 1.16 μM, control, *n* = 34) versus morphine-treated mice (EC50 = 0.21 ± 0.09, in vivo MS, *n* = 23) or naïve culture DRG neurons exposed to morphine (EC50 = 0.33 ± 0.02 μM, in vitro MS, *n* = 51). The shift towards lower concentration indicates that morphine potentiates the sensitivity of TRPM8 to menthol. Statistical analysis was performed using one and Two-Way ANOVA followed by Bonferroni post hoc test (**p < 0.01). Data are mean values, error bars indicate ±SEM

We then concentrated our work on TRPM8 since both cold and menthol responses were affected equally by morphine treatment. To determine the signaling pathways by which morphine was able to reduce TRPM8 desensitization, we tested the effect of morphine on cultured DRG neurons. As found with morphine-treated mice, in vitro exposure of neurons to morphine (500 nM, 16 h) increased both cold- and menthol-evoked depolarization and AP discharge (Fig. 3a and b**).** Importantly, application of the TRPM8 specific blocker AMTB (30 μM) was able to induce membrane potential hyperpolarization and block menthol-evoked AP discharge, as previously described [29] (Fig. 3a and b**)**. Strikingly, our in vitro chronification model corroborated our chronically treated animals as morphine exposure suppressed TRPM8 desensitization to cold or menthol and appeared to even potentiate TRPM8 responsiveness in a small proportion of cells. (Fig. 3d and e). In agreement with a sensitization of TRPM8 to cold or menthol following morphine treatment, the temperature-response curve of TRPM8 was shifted (~ 4 °C) towards warmer temperatures in both DRG neurons from morphine-sensitized animals and neurons from naïve mice exposed to morphine (Fig. 3f). Likewise, morphine induced a leftward shift of the dose-response curve of TRPM8 sensitivity to menthol (Fig. 3g). A similar pattern of sensitization was found in HEK cells transfected with TRPM8 + MOR and exposed to morphine overnight (Fig. S2a and b).

### Morphine-induced reduction in TRPM8 desensitization requires PKCβ activation

**Fig. 4:**
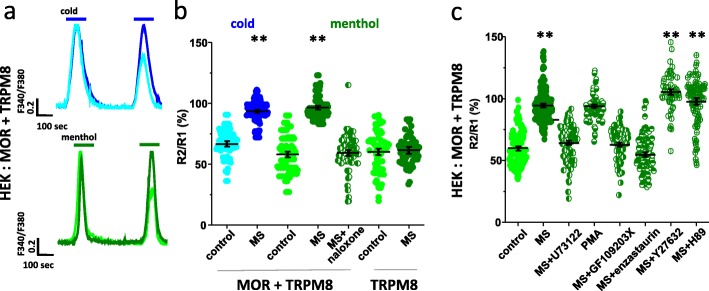
Reduction in activation-induced desensitization of TRPM8 is mediated by MOR and PKC. **a** Representative calcium imaging traces to two repeated applications of cold (20 °C, blue) or menthol (100 μM, green) in HEK cells transfected with TRPM8 + MOR, and exposed to morphine (MS, 0.5 μM) overnight. **b** Whisker plots showing the mean ratio (R2/R1) of the amplitude response to cold (66.97 ± 1.37% in control, light blue, vs 93.49 ± 0.95% in MS, dark blue; *n* = 85 and 103, respectively) or menthol (58.13 ± 1.66% in control, light green vs 96.58 ± 0.94%, in MS, dark green, and 59.4 ± 1.74%, half symbols, with MS + naloxone; *n* = 93, 111 and 71, respectively in HEK cells transfected with MOR+TRPM8 or in the absence of MOR (60.2 ± 2.17% (*n* = 66) and 61.58 ± 1.52% (*n* = 60) in control conditions and after MS overnight, respectively). **c** Whisker plots showing the mean ratio (R2/R1) of the amplitude response to menthol in transfected HEK cells exposed to the different conditions of treatment: control (59.93 ± 1.41%, *n* = 100); MS (94.45 ± 0.98%, *n* = 185), MS + PLC inhibitor U73122 (1 μM; 64.12 ± 1.66%, *n* = 82), PKC activator PMA (1uM; 94.9 ± 1.04, *n* = 85), MS + PKC blocker GF109203X (3 μM; 62.62 ± 1.7%, *n* = 66), MS+ PKCβ blocker enzastaurin (1 μM; 54.58 ± 1.69%, *n* = 85); MS + ROCK inhibitor Y27632 (1 μM; 105.27 ± 2.01%, *n* = 55) and MS + PKA inhibitor H89 (100 nM; 97.46 ± 1.9%, *n* = 92). Error bars indicate ±SEM. Statistical analysis was performed using the Kruskal-Wallis ANOVA followed by the Dunn-Bonferroni post hoc test (**p < 0.05)

We next interrogated the MOR signaling pathway that regulated TRPM8 desensitization. In transfected HEK cells, sustained application of morphine negated the TRPM8 desensitization as observed in neurons (Fig. 4a). Reduction of TRPM8 desensitization by morphine depended on the expression of MOR and was lost upon application of its competitive antagonist Naloxone. These data suggested a selective MOR-mediated signaling pathway that regulated channel desensitization (Fig. 4b). In addition, the effect of morphine on TRPM8 desensitization required long lasting treatment, as acute exposure failed to prevent menthol- or cold-evoked desensitization of TRPM8 measured by whole cell patch clamp (Fig. S3a) or calcium imaging (Fig. S3b). Strikingly, the effect appeared to be triggered by morphine-bound MOR signaling since [D-Ala2, N-MePhe4, Gly-ol]-enkephalin (DAMGO), a highly specific MOR agonist, failed to inhibit TRPM8 desensitization (Fig. S2c). Further investigation with other Gq- or Gi-coupled receptors showed that sustained activation of the protease activated receptor (PAR2), the α2 or α1 adrenergic receptor, or the cannabinoid receptor CB1 also failed to prevent TRPM8 desensitization in response to their specific agonists (Fig. S2d). Altogether, our findings reveal the sensitization of TRPM8 by virtue of morphine-induced activation of MOR.

MOR is coupled to the Gαi/o class of Gα proteins. While activation of MOR by DAMGO induces β-arrestin (βARR) recruitment and receptor internalization, morphine was shown to exhibit a different signaling profile with preference to PLC activation, mitogen-activated protein kinase (MAPK) and PKC-dependent desensitization [4, 30, 31]. PLC signaling mediates hydrolysis of phosphatidyl inositol 4,5-phosphate 2 (PIP2), a membrane-associated phospholipid that maintains the open probability of TRPM8 channels [23, 32], and generates diacylglycerol (DAG) [33, 34]. We next used pharmacological blockers to test the contribution of this pathway on TRPM8 desensitization. Morphine-induced inhibition of TRPM8 desensitization was reversed by using the PLC inhibitor U73122 (1 μM), the broad spectrum PKC inhibitor GF109203X (3 μM) as well as by the specific PKCβ blocker enzastaurin (1 μM) (Fig. 4c). In contrast, blocking PKA or ROCK was not able to prevent MOR-induced reduction in TRPM8 desensitization. Importantly, in the absence of morphine, activation of PKC with an acute PMA treatment was able to prevent desensitization of TRPM8 (Fig. 4c). These results suggest that activation of MOR by morphine stimulates a MOR-PLC-PKCβ signaling that dictates TRPM8 desensitization.

### Site-specific regulation of TRPM8 at Ser 1040 and 1041 mediates morphine-induced reduction in TRPM8 desensitization

**Fig. 5:**
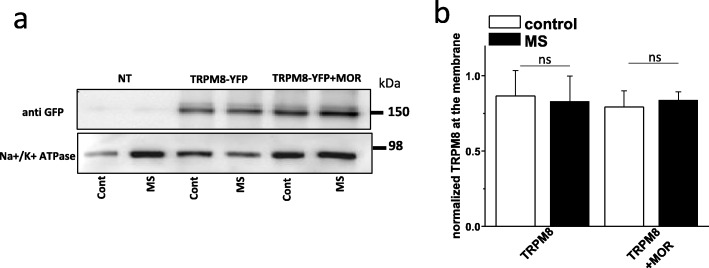
Activation of MOR by morphine does not change TRPM8 trafficking. **a** Western blot of surface biotinylated YFP-tagged TRPM8 in HEK cells. Top, surface biotinylation of untransfected cells, TRPM8-YFP or TRPM8-YFP + MOR transfected cells exposed to vehicle control or morphine (MS); bottom, western blot of plasma membrane Na+/K+ ATPase marker used as control. **b** Bar graph summarizing the biotinylation results illustrated in (**a**). Blots are representative, data are means ± SEM of the TRPM8 band intensity signal normalized to the Na+/K+ ATPase, from 4 independent experiments (unpaired t-test)

Phosphorylation of ion channels by PI3K or PKC is known to regulate cell surface expression of ion channels in sensory neurons [30, 35] and opioids have been proposed to modulate thermosensation by internalizing the TRPM8 channel [18]. To test whether MOR sensitized channel function by modulating channel trafficking, we used biotinylation assay in HEK cells to assess the cell surface expression of TRPM8 following morphine treatment. In the absence or presence of the MOR, insertion of TRPM8-YFP at the plasma membrane was unaffected by morphine treatment (Fig. 5a and b). These data confirm that morphine augments TRPM8 sensitivity to cold or menthol, without altering its expression at the cell surface.

**Fig. 6:**
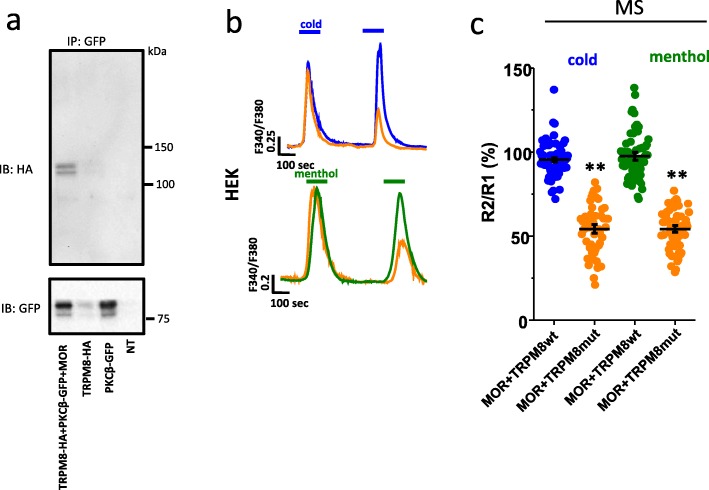
MOR enhances TRPM8 sensitivity to cold and menthol through PKCβ-dependent phosphorylation. **a** Representative co-immunoprecipitation of GFP-tagged PKCβ with the full-length HA-tagged TRPM8 in HEK cells for different conditions of transfection (*n* = 3 independent experiments). Lower panel, input fraction immunoblotted with HA antibody (note the unspecific lower band that appears in the untransfected condition. The upper band is only found in condition of TRPM8-HA transfection). Middle panel, the imunoprecipitated GFP- PKCβ was detected with a GFP antibody. Upper panel shows the western blot of the TRPM8-HA western blot of the immunoprecipitated fraction. **b** Representative calcium imaging traces of TRPM8 WT and TRPM8SS1040-1041AA mutant (orange) co-transfected with MOR and stimulated by two repeated applications of cold (blue) or menthol (green) after MS application. Note the absence of desensitization with the WT but not the mutant channel. **c** Whisker plots showing the mean ratio (R2/R1) of the amplitude response to cold or menthol, represented in B, in HEK cells transfected with MOR and TRPM8 WT or TRPM8 mut (SS1040-1041AA mutant) (orange) after morphine treatment (wt: 95.46 ± 1.39% (blue) vs. mutant: 54.16 ± 2.03% (orange), *n* = 61 and 50 respectively, in response to cold; wt: 97.5 ± 1.79% (green) vs. mutant: 54.24 ± 1.51% (orange), *n* = 62 and 60, respectively, in response to menthol). Statistical analysis was performed using One-Way ANOVA followed by Bonferroni post hoc test (**p < 0.01). Error bars indicate ±SEM

In order to examine how MOR-PKCβ signaling was able to regulate TRPM8 desensitization, we asked if TRPM8 associated with the kinase using co-immunoprecipiation. As shown in Fig. 6a, PKCβ-GFP immunoprecipitated a TRPM8-HA channel in transfected HEK cells, indicating the existence of a TRPM8- PKCβ signaling complex that could respond to MOR activation. Due to a lack of a specific P-Ser/Thr TRPM8 antibody we were unable to examine the level of phosphorylation of TRPM8 in response to morphine (see discussion), however, we examined the phosphorylation consensus amino acid sequence for PKC phosphorylation (RXXS/TXRX; where X is any amino acid) using PhosphoSitePlus. This *in sillico* analysis revealed several possible phosphorylation sites with only two highly conserved consensus motifs: one at Ser850 (^843^LIHIFTVSRNLGPKI^857^) at the end of the S4 segment, the second in the C terminus (^1034^KEKNMESSVCCFKNE^1048^) of TRPM8. We focused on the C-terminal region of the channel that contains the TRP domain involved in menthol binding and cold sensing [36–39]. Serines were replaced with alanines at sites 1040 and 1041. Expression of channel mutants in HEK cells did not indicate alterations in the current-voltage relationship, and the current density evoked by menthol, compared to wild type TRPM8 (Fig. S2e). Nevertheless, expression of the mutant channel completely eliminated the morphine-induced sensitization of both cold and menthol-evoked calcium response (Fig. 6b and c). These results strongly suggest that activation of MOR by morphine elicits a PKCβ-mediated phosphorylation of TRPM8 at Ser 1040 and 1041, which in turn prevents channel desensitization.

## Discussion

**Fig. 7:**
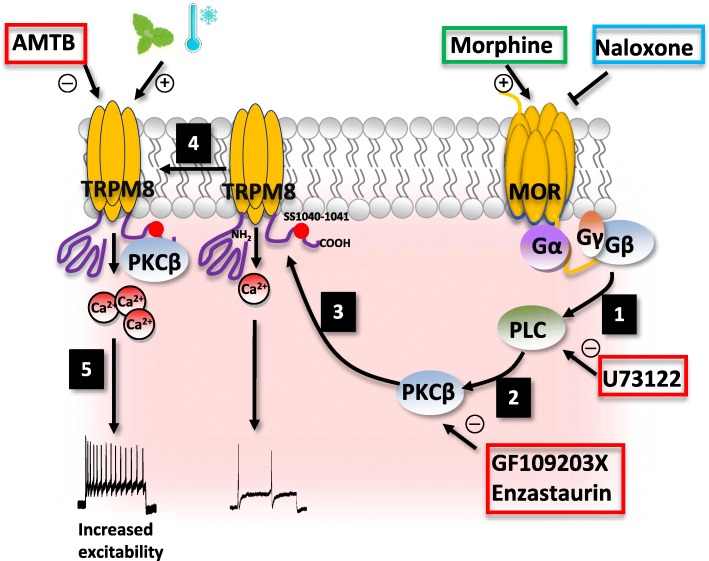
Schematic representation of site-specific regulation of TRPM8 by MOR-PKCβ signaling. Sustained morphine treatment acting on MOR induces PLC activation (1) which regulates PKCβ activity (2). The activated PKCβ phosphorylates the C terminus domain of the TRPM8 channel at S1040 and S1041 (3). This leads to a reduction of activity-induced channel desensitization (4). Both, increase in excitability of TRPM8-expressing neurons and reduction in activity-induced desensitization promotes morphine-induced cold hypersensitivity that is associated with chronic opioid treatment

Cold hypersensitivity is an important behavioral manifestation of chronic morphine treatment. Here we report that morphine-induced cold hyperalgesia in mice is associated with 1) an increase in neuronal excitability of TRPM8-expressing DRG neurons and 2) a loss of TRPM8 desensitization evoked by cold or menthol (see Fig. 7). We showed that mice chronically exposed to morphine exhibit cold hyperalgesia, and neurons isolated from these mice are more excitable than neurons from naïve mice. Importantly, we found that morphine enhances the sensitivity of TRPM8 to cold or menthol and reduces channel desensitization. These changes in TRPM8 activity seem to account for the increased neuronal excitability induced by morphine as the TRPM8 blocker AMTB was able to hyperpolarize menthol sensitive neurons and inhibit AP discharge. We also found that prolonged activation of MOR by morphine contributes to the more positive resting membrane potential and increased both the frequency of spontaneous action potential and the AP firing in response to menthol. Despite the hyperpolarizing effect of AMTB, we thus cannot rule out that alterations in the activity of voltage gated calcium or potassium channels occur following morphine treatment. Overall, alterations in voltage-dependent ionic conductances leading to enhanced excitability, along with changes in TRPM8 activity, may together promote cold hypersensitivity induced by morphine.

Both morphine and endogenous enkephalin peptides are potent analgesics that act on MOR, however morphine displays partial agonism at the MOR and predominantly exerts both analgesic effects and undesirable effects through receptor activation [40]. This was confirmed by our experiments showing that naloxone suppresses the increased excitability of cold nociceptors. In addition, absence of the MOR in HEK cells blocked the loss of TRPM8 desensitization induced by morphine. It still remains unclear to what degree morphine engages distinct signaling effectors in neurons, yet there is accumulating evidence that morphine promotes the recruitment of βARR2 preferentially over βARR1 but poorly internalizes MOR [40]. Furthermore, while morphine-bound MOR was thought to be biased toward cAMP inhibition over trafficking and pERK activation, Stoeber et al. reported that morphine induced the activation of Golgi-localized opioid receptors, suggesting intracellular sites of MOR activation by morphine compared to enkephalin peptides [41]. This agonist specific activation pattern may also explain why DAMGO was not able to elicit a loss of TRPM8 desensitization as found with morphine.

The activity of the thermosensor TRPM8 in primary sensory neurons is unique in that it senses cool temperatures but also mediates cold-induced analgesia [10–12]. TRPM8 was previously found to be involved in the development of cold hyperalgesia following chronic morphine administration, although the underlying mechanisms of this effect involved an upregulation of the channel expression [19]. Shapovalov et al. found a distinct mechanism of TRPM8 regulation in rat DRG neurons with morphine administration leading to an increase in TRPM8 internalization [18]. In contrast to what was reported by Gong et al. [19] we did not observe an increase in TRPM8 mRNA expression in response to morphine. In addition, assessment of plasma membrane biotinylated fraction did not reveal an alteration in TRPM8 surface expression. These negative results support the idea that regulation of TRPM8 intrinsic activity rather than change in channel expression account for the altered thermosensitivity observed upon escalating dose of morphine. Furthermore, the expression of the receptor at the mRNA or protein level has been reported to be relatively stable, with a few exceptions, following morphine treatment [42, 43]. Accordingly, despite a central role of MOR in the development of morphine-induced cold hyperalgesia, we did not see a change in the expression of MOR mRNA in our experimental conditions.

Both cold and menthol sensitivities were found to be enhanced by morphine. Previous studies described menthol as being promiscuous in its targets with the possibility of modulating the activity of other thermosensors (TRPA1, TRPV3, TRPC5, Nav1.9 and two pore potassium channels) [11, 26, 28, 44]. Yet, menthol inhibits the activity of most of these channels at the concentrations used, including TRPA1 which was considered to be a noxious cold sensor [11, 26–28, 44]. At the molecular level, we found that MOR activation by morphine leads to a PKCβ-induced reduction of TRPM8 desensitization likely via phosphorylation of the two Ser residues 1040 and 1041. While MOR acutely inhibits cAMP generation via Gi/o proteins, blocking PKA with H89 was not able to prevent TRPM8 regulation. In addition, we examined the RhoA/ROCK pathway that is involved in many cellular functions, including thermal hyperalgesia [45]. Our calcium signaling experiments using the Y27632 suggests that ROCK signaling does not contribute to TRPM8 regulation downstream of MOR.

The reduction in channel desensitization may be relevant to the transmission of cold signaling in primary afferent neurons and our findings suggest a crucial role of TRPM8 phosphorylation in this process. Activation of MOR by morphine was previously found to activate PLC and ERK [4, 5]. The PLC signaling cascade mediates hydrolysis of PIP2, which, in turn, inhibits and desensitizes TRPM8 [23, 32]. In addition, morphine-mediated PLC activation generates DAG that recruits and activates PKC at the membrane. Previously, Abe et al. showed that PKC (conventional α and/or β1 subtype) induced menthol-evoked desensitization of TRPM8 [46]. However, none of the putative serine/threonine PKC phosphorylation sites (T312A, S319A, S541A, and T556A) examined in this study appeared to be involved in channel modulation and the authors concluded that PKC had an indirect effect on TRPM8 modulation [46]. Premkumar et al. showed that bradykinin-induced activation of PKC caused TRPM8 dephosphorylation and downregulation in rat neonatal DRG neurons [47]. Overall, the contribution of PKC in TRPM8 regulation has remained unclear since the DAG analogue OAG did not inhibit TRPM8 activity [23], and PKC inhibitors failed to block desensitization [23] and menthol-induced currents [24]. Most of the observed effects may likely be attributable to PIP2 hydrolysis, which is required to maintain TRPM8 activity [13]. Our data suggest that activation of PKC, in the presence of MOR expression, prevented channel desensitization. Several experimental differences could explain this discrepancy with the previous findings, including the Ca2+ free solution used in Abe’s study or the absence of MOR expression. In addition, Premkumar and coll. conducted their electrophysiological recordings in Xenopus oocytes and cultured neonatal DRGs, where the lower expression of MOR might also explain the difference in TRPM8 desensitization properties. Finally, as we showed that PKCβ co-immunoprecipitates with TRPM8 in the presence of MOR, it is possible that MOR expression optimizes the anchoring of PKCβ to the channel, and thus facilitates its phosphorylation at Ser 1040 and 41.

Our experiments indicate a role of PKC downstream of morphine-induced MOR activation. Application of a PKC blocker was able to reverse the loss of TRPM8 desensitization produced by morphine. In silico identification of PKC consensus sites and site-directed mutagenesis of Ser 1040 and 1041 completely blocked the morphine-induced inhibition of TRPM8 desensitization. The site of the mutation is in the vicinity of the S6-TRP box linker which seems to be a central determinant in channel gating by voltage and menthol [38]. This C-terminal region is also important in cold sensing and has several PIP2 binding sites involved in channel gating [36–39]. Using co-immunoprecipiation we showed that PKCβ was able to associate with TRPM8 in HEK cells, however we were not able to demonstrate whether TRPM8 was phosphorylated by PKCβ. We recognize that such data set would link our pharmacological experiments in Fig. 4 and the site directed mutagenesis study that identifies Ser 1040/41 (Fig. 6), however there are no phospho Ser/Thr TRPM8 antibodies available to perform immunostaining or western blotting analysis in the chronic morphine model. Moreover, a Pan-Ser/Thr antibody can be used on immunoprecipitated TRPM8, but since a very small proportion of neurons express TRPM8, the likelihood of measuring an increase in Ser/Thr phosphorylation of TRPM8 channels downstream of morphine signalling is low. Furthermore, Ser/Thr phosphorylation may only occur on membrane-bound TRPM8, in proximity of the MOR, rather than whole-cell immunoprecipitated TRPM8 channels. Ongoing studies in our lab using mass spectrometry analysis will hopefully clarify whether phosphorylation of S1040 and 1041 can be unequivocally accepted. Finally, the PKC is a large family of serine/threonine kinases, composed of eleven different enzymes. Given that PKCε, α and β are abundant in small peptidergic and non-peptidergic DRG neurons, and based on the transcriptomic studies indicating the expression of PKCβ in the TRPM8 population [48, 49], future work will be warranted to examine whether PKCβ expression is altered in response to chronic morphine, and if there is a shift in its pattern of expression among the DRG neuron population.

In summary, our data indicates that chronic morphine treatment enhances the sensitivity to cooling temperature by inducing de novo responsiveness to cold and dampening TRPM8 desensitization in DRGs. This process occurs through PKCβ phosphorylation at newly identified phosphorylation sites of TRPM8. Therefore, blocking PKCβ-mediated regulation of TRPM8 could constitute a valuable strategy to attenuate opioid-induced cold hyperalgesia.

## Supplementary information

**Additional file 1: Figure S1.** Morphine treatment increases the evoked neuronal activity. (a) Representative AP discharges evoked by 100, 200 and 300 pA current injections (1 s) in DRG neurons from control (black) and morphine-treated (red) animals (2.75 ± 0.6 Hz in control (black) vs 16.00 ± 3.5 Hz (red) evoked by the injection of 300 pA of current, *n* = 10 and 9, respectively. (b) Mean values of the data presented in (a). (c) Expression of TRPM8, MOR, TREK-1 and TRAAK mRNA in total DRGs harvested from control (black) and morphine treated mice (red). (d) Mean ratio (R1/baseline) of the amplitude response to menthol or cold stimulation in control (3.51 ± 0.23, light green; 3.29 ± 0.15 light blue), after overnight morphine treatment in DRG culture (3.47 ± 0.26, dark green; 3.6 ± 0.16, dark blue), or chronic morphine treatment in mice (3.18 ± 0.27, dark green; 3.28 ± 0.32, dark blue); *n* = 32, 30 and 30 for menthol and 30, 31 and 30 for cold. **Figure S2.** Morphine potentiates TRPM8 sensitivity to cold and menthol in transfected HEK cells. (a) Temperature-response curve measured by calcium imaging during cooling ramp on HEK cells transfected with TRPM8 and MOR from untreated (open symbols) versus morphine-treated cells (closed symbols). Sensitization of the temperature-response relationship is indicated by the shift towards warmer temperature; median temperature value 21.64 ± 0.65 °C, control vs 25.41 ± 0.58 °C, MS; *n* = 52 and 91, respectively). (b) Dose-response curve evoked by menthol, measured by calcium imaging on HEK cells transfected with TRPM8 and MOR from untreated (open symbols) versus morphine-treated cells (closed symbols). The shift towards lower concentration indicates that morphine potentiates the sensitivity of TRPM8 to menthol; EC50 = 11.44 ± 3.4 μM, control vs EC50 = 0.59 ± 0.02 μM, MS; *n* = 91 and 76, respectively. (c) R2/R1 amplitude ratio of two repeated applications of menthol in HEK cells transfected with TRPM8 and MOR and exposed to DAMGO (0.05 μM) overnight (63.84 ± 1.81%, *n* = 46 vs. 60.18 ± 1.47% control, *n* = 58). (d) R2/R1 amplitude ratio of two repeated applications of menthol in HEK cells transfected with TRPM8 and G protein-coupled receptors (PAR2, α1AR or α2AR, CB1) and exposed to their specific agonists (61.25 ± 7.13%, *n* = 25 for 1 μM 2fLI, 58.46 ± 5.45%, *n* = 32 for 1 μM clonidine, 58.25 ± 4.51%, *n* = 29 for 1 μM phenylephrine, and 67.42 ± 2.96%, *n* = 34 for 0.5 μM CBD) overnight. (e) Current density -Voltage curves in control or menthol stimulation in HEK cells transfected with TRPM8-WT (black) and SS1040-1041AA mutant (orange). Data are mean values, error bars indicate ±SEM. Statistical analysis was performed using unpaired t-test, One and Two-Way ANOVA followed by Bonferroni post hoc test (**p < 0.01). **Figure S3.** Acute treatment with morphine did not prevent TRPM8 desensitization in response to cold and menthol. (a) Representative whole cell current-clamp recording of the menthol-evoked current at + 100 mV and − 100 mV from a holding potential of 0 mV, in response to an acute (5 min) treatment with morphine (1 μM). (b) R2/R1 amplitude ratio of two repeated applications of menthol (green) or cold (blue) after an acute morphine treatment, in HEK cells transfected with TRPM8 and MOR.

**Additional file 2.**

## Data Availability

The data used in our study are available from the authors on reasonable request.

## Abbreviations

TRPM8: Transient Receptor Potential Melastatin 8
MOR: Mu opioid receptor
MS: Morphine sulphate
PKC: Protein kinase C
PLC: Phospholipase C
ROCK: Rho-associated protein kinase
GAPDH: Glyceraldehyde 3-phosphate dehydrogenase; α1AR or α2AR, alpha adrenergic receptor
