# Supplementary figures and images for: Chronic morphine regulates TRPM8 channels via MOR-PKCβ signaling
Source: Mol Brain. 2020 Apr 14;13:61. doi: 10.1186/s13041-020-00599-0 (PMC7155267; doi:10.1186/s13041-020-00599-0)

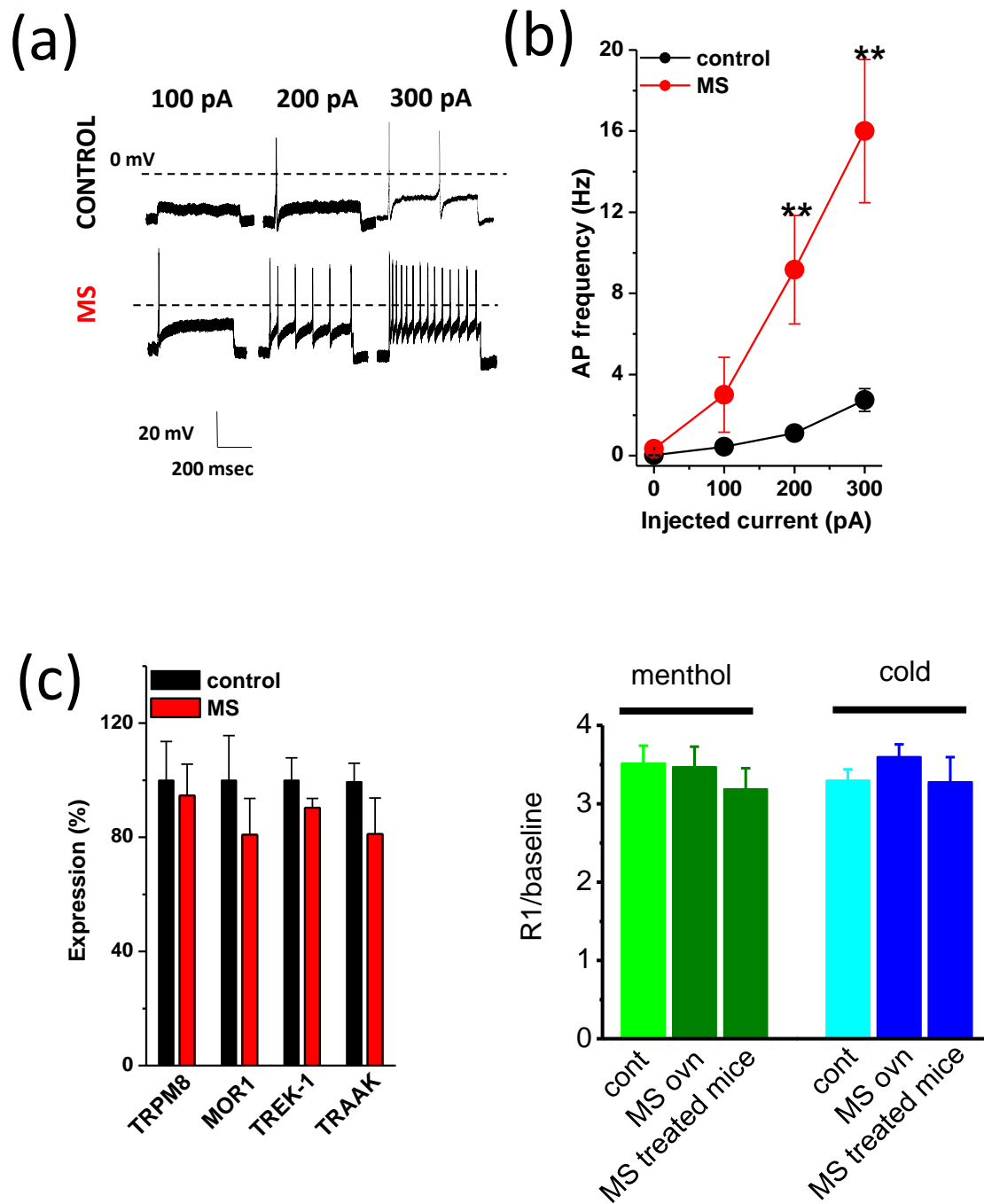

Figure S1

(a)

HEK: MOR+TRPM8

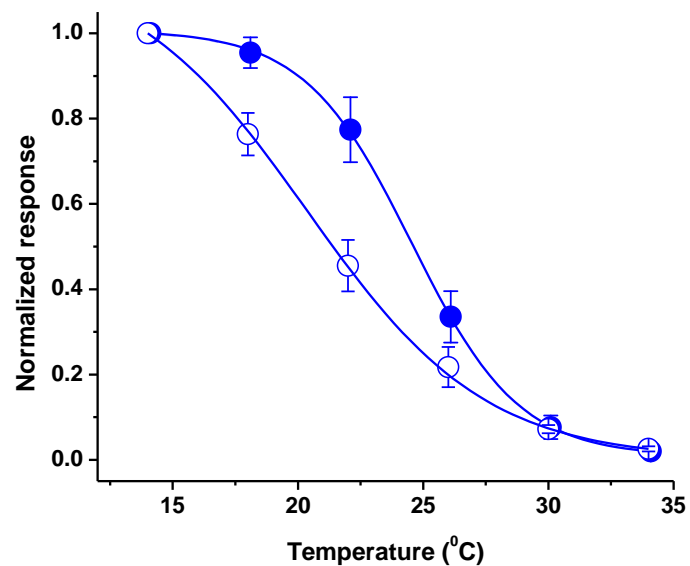

(b)

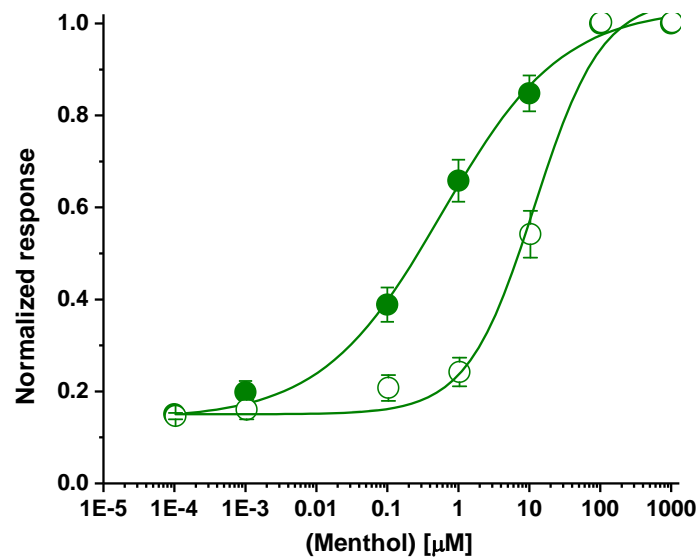

(c)

menthol

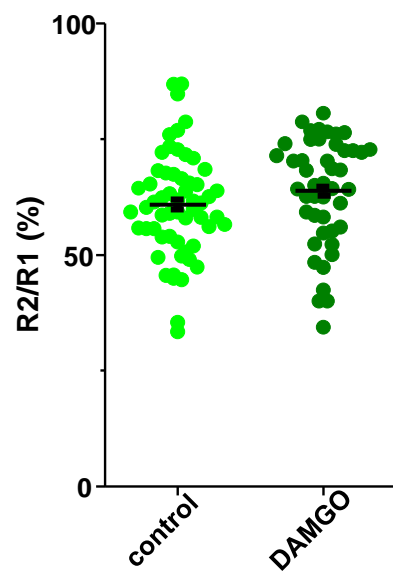

(d)

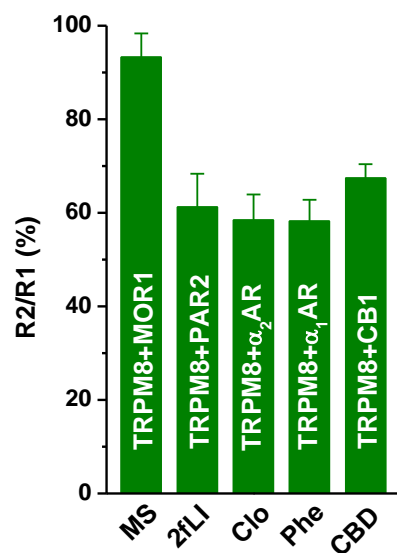

(e)

HEK: MOR+TRPM8mut

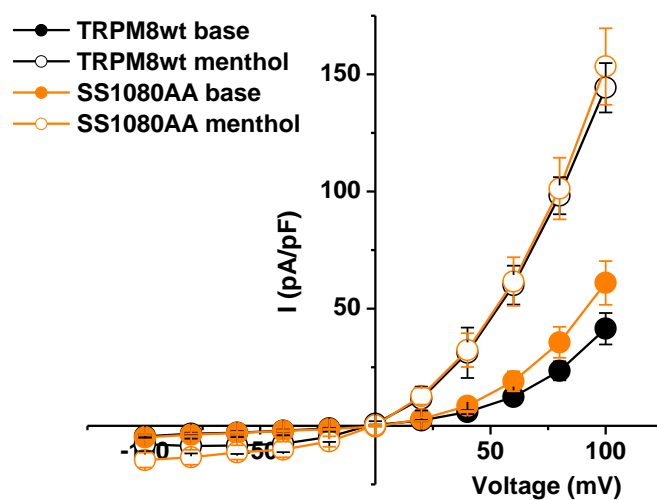

Figure S2

# HEK: TRPM8+MOR

(a)

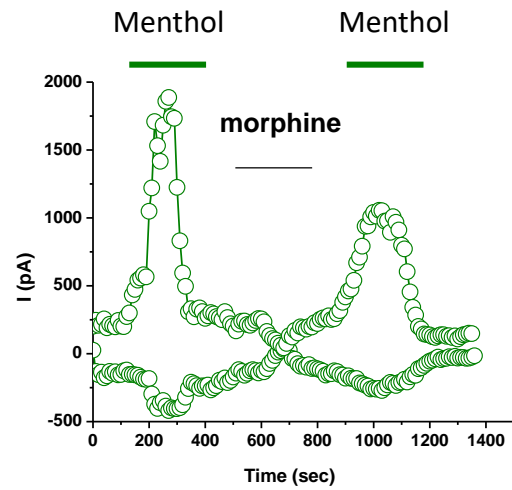

(b)

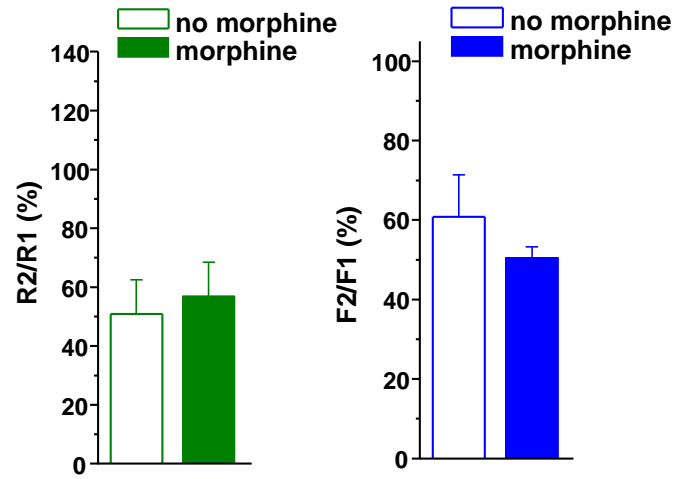

Figure S3

Supplement: Supplementary file 1 — Additional file 1: Figure S1. Morphine treatment increases the evoked neuronal activity. (a) Representative AP discharges evoked by 100, 200 and 300 pA current injections (1 s) in DRG neurons from control (black) and morphine-treated (red) animals (2.75 ± 0.6 Hz in control (black) vs 16.00 ± 3.5 Hz (red) evoked by the injection of 300 pA of current, n = 10 and 9, respectively. (b) Mean values of the data presented in (a). (c) Expression of TRPM8, MOR, TREK-1 and TRAAK mRNA in total DRGs harvested from control (black) and morphine treated mice (red). (d) Mean ratio (R1/baseline) of the amplitude response to menthol or cold stimulation in control (3.51 ± 0.23, light green; 3.29 ± 0.15 light blue), after overnight morphine treatment in DRG culture (3.47 ± 0.26, dark green; 3.6 ± 0.16, dark blue), or chronic morphine treatment in mice (3.18 ± 0.27, dark green; 3.28 ± 0.32, dark blue); n = 32, 30 and 30 for menthol and 30, 31 and 30 for cold. Figure S2. Morphine potentiates TRPM8 sensitivity to cold and menthol in transfected HEK cells. (a) Temperature-response curve measured by calcium imaging during cooling ramp on HEK cells transfected with TRPM8 and MOR from untreated (open symbols) versus morphine-treated cells (closed symbols). Sensitization of the temperature-response relationship is indicated by the shift towards warmer temperature; median temperature value 21.64 ± 0.65 °C, control vs 25.41 ± 0.58 °C, MS; n = 52 and 91, respectively). (b) Dose-response curve evoked by menthol, measured by calcium imaging on HEK cells transfected with TRPM8 and MOR from untreated (open symbols) versus morphine-treated cells (closed symbols). The shift towards lower concentration indicates that morphine potentiates the sensitivity of TRPM8 to menthol; EC50 = 11.44 ± 3.4 μM, control vs EC50 = 0.59 ± 0.02 μM, MS; n = 91 and 76, respectively. (c) R2/R1 amplitude ratio of two repeated applications of menthol in HEK cells transfected with TRPM8 and MOR and exposed to DAMGO (0.05 μM) ove [file 13041_2020_599_MOESM1_ESM.pdf]
