# Supplementary material for: Chronic morphine regulates TRPM8 channels via MOR-PKCβ signaling
Source: Mol Brain. 2020 Apr 14;13:61. doi: 10.1186/s13041-020-00599-0 (PMC7155267; doi:10.1186/s13041-020-00599-0)
Supplement: Supplementary file 2 — Additional file 2. [file 13041_2020_599_MOESM2_ESM.pdf]

Gene	Forward (3’-5’)	Reverse (5’-3’)
RPLP	AAGAACACCATGATGCGCAAG	TTGGTGAACACGAAGCCCA
TRPM8	CCCGAGCAGTGGAGTTGTTC	GCTTCGCAGGAGTAGACCAG
MOR	GAGCCACAGCCTGTGCCCT	CGTGCTAGTGGCTAAGGCATC
TRAAK	CAATCCCAGCCTGGAAGGTT	CAGTGGTCACTGCACCTG
TREK-1	TATACTGCAGGAGTGGCGG	GCCTCGGTTTGGAGTTCTGA

Suppl Table 1
